# Getting a grasp on action-specific scaling: A response to Witt (2017)

**DOI:** 10.3758/s13423-018-1511-0

**Published:** 2018-09-20

**Authors:** Elizabeth S. Collier, Rebecca Lawson

**Affiliations:** Department of Experimental Psychology, University of Liverpool, Eleanor Rathbone Building, Bedford Street South, Liverpool, L69 7ZA UK

**Keywords:** Vision. Action-specific perception. Grasping. Action capacity

## Abstract

Can higher level cognition directly influence visual spatial perception? Many recent studies have claimed so, on the basis that manipulating cognitive factors (e.g., morality, emotion, action capacity) seems to directly affect perception. However, Firestone and Scholl (*Behavioral and Brain Sciences*, *39*, 1–77, 2016) argued that such studies often fall prey to at least one of six pitfalls. They further argued that if an effect could be accounted for by any of these pitfalls, it is not a true demonstration of a top-down influence of cognition on perception. In response to Firestone and Scholl (*Behavioral and Brain Sciences*, *39*, 1–77, 2016), Witt (*Psychonomic Bulletin & Review*, *24*(4), 999–1021, 2017) discussed four action-specific scaling effects which, she argued, withstand all six pitfalls and thus demonstrate true perceptual changes caused by differences in action capacity. Her third case study was the influence of apparent grasping capacity on perceived object size. In this article, we provide new interpretations of previous findings and assess recent data which suggest that this effect is not, in fact, perceptual. Instead, we believe that many earlier studies showing this effect are subject to one or more of the pitfalls outlined by Firestone and Scholl (*Behavioral and Brain Sciences*, *39*, 1–77, 2016). We substantiate our claims with recent empirical evidence from our laboratory which suggests that neither actual nor perceived grasping capacity directly influence perceived object size. We conclude that studies manipulating grasping capacity do not provide evidence for the action-specific account because variation in this factor does not directly influence size perception.

**Electronic supplementary material:**

The online version of this article (10.3758/s13423-018-1511-0) contains supplementary material, which is available to authorized users.

## Introduction

Understanding how the mind is organised is central to theories of spatial perception, and this involves understanding which factors contribute to what is perceived (Firestone & Scholl, 2016; Witt, 2017). In this article, we provide an independent contribution to the ongoing debate concerning whether action capacity directly influences visual perception (for reviews, see Firestone, 2013; Philbeck & Witt, 2015; Proffitt, 2013; Proffitt & Linkenauger, 2013; Witt, 2011, 2017; Witt & Riley, 2014). First, we would like to outline our position in the action-specific debate. When we began investigating the claims of the action-specific account, we were not active in the wider debate. Rather, we found the idea that action capacity could influence visual perception intriguing. We therefore investigated the mechanism purported to underlie these effects, and whether comparable effects to those obtained for vision would be found for our sense of active touch (haptics). In pursuit of this, we ran a series of studies (Collier & Lawson, 2017a) based on the findings of Linkenauger, Witt, and Proffitt (2011), which suggested that apparent grasping capacity directly influenced perceived object size. We failed to replicate the effect reported by Linkenauger, Witt, and Proffitt (2011, Experiment 2), and we sought to understand why. As we further investigated the apparent influence of grasping capacity and hand size on perceived object size (Collier & Lawson, 2017b, 2018), we became increasingly convinced that these effects are not caused by a true perceptual change as the action specific account claims. Instead, we now believe that this effect can be explained by other factors including experimental demand characteristics, strategies such as using the size of familiar objects to anchor estimates, and visual illusions. These alternative explanations are not compatible with the action-specific account (Firestone, 2013).

The present paper is a direct response to a recent review paper by Witt (2017), a proponent of the action-specific account. In her review, Witt provides a detailed discussion of four empirical case studies. She claimed that each case provided evidence for action-specific effects that did not fall prey to any of the pitfalls outlined by Firestone and Scholl (2016) which could provide an alternative explanation of action-specific effects. Her third case study focussed on the claim that grasping capacity exerts a direct influence on perceived object size. In our response here, we discuss our own empirical data which provides evidence against this claim. We also offer a critical evaluation of the arguments made in Witt’s (2017) third case study and new interpretations of previous results claiming to support the action-specific account. We conclude that the evidence supporting Witt’s (2017) claim that grasping capacity directly influences perceived object size is not reliable.

## Cognition and perception

What is meant by *perceiving* something? Phenomenologically, this is relatively clear: we can *see* the yellowness of a banana, *hear* the melody in our favourite song, and *feel* a breeze against our skin. In each of these cases, incoming signals from external stimulation of the relevant sensory system give rise to a perceptual experience. These experiences seem distinct from, for example, *knowing* the price of a banana in your local shop, *recalling* when you first heard your favourite song, or *imagining* how pleasant a breeze might be on a warm day, all of which could be considered examples of cognition. To borrow from Firestone and Scholl (2016), the distinction between perception and cognition usually seems “natural and robust” (p. 1).

Despite the phenomenologically simple distinction between perception and cognition, there has been fierce debate as to whether our perceptual experiences are truly independent of cognitive influence (Firestone & Scholl, 2016, 2017; Proffitt & Linkenauger, 2013; Pylyshyn, 1999; Vetter & Newen, 2014). Some evidence appears to support the idea that perception is directly influenced by cognitive factors in a nontrivial way. For example, it has been suggested that desires (e.g., Balcetis & Dunning, 2010; Stokes, 2012), morality (e.g., Gantman & Van Bavel, 2014), and emotions (e.g., Stefanucci & Proffitt, 2009) can literally change what is perceived. Such effects would indicate *cognitive penetrability—*the notion that what we perceive can be directly altered, top-down, by cognitive states. This, in turn, would challenge the claim that perception is *cognitively impenetrable* (Pylyshyn, 1999; Firestone & Scholl, 2016). If perception was found to be penetrable by cognitive factors, then our current understanding of perception would need a drastic overhaul (Firestone, 2013; Firestone & Scholl, 2016), and so this is an important issue to address.

There has been debate about what kinds of effects count as examples of cognitive penetrability (Firestone & Scholl, 2016; Stokes, 2012; Witt, 2017). Since it is difficult to convincingly state whether an effect demonstrates cognitive penetrability, Firestone and Scholl (2016) proposed that, instead, researchers should consider what does *not* constitute cognitive penetrability. They outlined six *pitfalls* which, they claimed, explained nearly all apparent examples of cognitive penetrability. They argued that if an effect could be explained by one or more of these pitfalls, then it should not be considered to provide evidence for cognitive penetrability in its strongest sense. The pitfalls are:

1. Only confirmatory predictions were tested; no attempt was made to produce disconfirmatory evidence.
2. Postperceptual judgements were measured, rather than online perception.
3. Effects could be explained by experimental demand and response bias.
4. Effects could be explained by variation in low-level perceptual features.
5. Effects could be caused by changes in the focus of attention.
6. Effects could be due to changes in memory and recognition.

## The action-specific account of perception

An increasing body of evidence has been claimed to support the hypothesis that what we see is scaled according to the action capabilities of our body. Proffitt and Linkenauger (2013) argued that this scaling may relate to variation in energetic expenditure and effort, as well as differences in performance success. Examples relating to energy expenditure and effort include the findings that hills were estimated as steeper when observers were fatigued or wore a heavy backpack (Bhalla & Proffitt, 1999; Proffitt, Bhalla, Gossweiler, & Midgett, 1995), and after participants consumed a sugar-free compared to a sugary beverage (Schnall, Zadra, & Proffitt, 2010). In addition, underwater targets were estimated as closer when people wore flippers which made swimming easier (Witt, Schuck, & Taylor, 2011). Examples resulting from differences in performance ability include the findings that putting holes and softballs were estimated as larger (Witt, Linkenauger, Bakdash, & Proffitt, 2008; Witt & Proffitt, 2005) and tennis balls were estimated as slower (Witt & Sugovic, 2010) by more successful players of the relevant sport.

It has also been suggested that what we see may scale according to the functional morphology of our body (Linkenauger, Ramenzoni & Proffitt, 2010; Linkenauger, Witt, & Proffitt, 2011; Proffitt & Linkenauger, 2013). For example, observers estimated targets to be nearer after reaching to them with a tool which increased their maximum reach and hence made the targets reachable (Witt, Proffitt, & Epstein, 2005). Also, door-like apertures were estimated as narrower when observers held a horizontal rod that was wider than their body (Stefanucci & Geuss, 2009). In a final example that we will consider in depth in the present paper, Linkenauger, Witt, and Proffitt (2011, Experiment 2) reported that right handers underestimated the size of objects they intended to grasp with their right hand relative to objects they intended to grasp with their left hand. Right handers perceive their right hand as larger than their left hand, and they also believe that it can grasp larger objects (Collier & Lawson, 2017a; Linkenauger, Witt, Bakdash, Stefanucci, & Proffitt, 2009; Linkenauger, Witt, & Proffitt, 2011). Based on this finding, Linkenauger, Witt, and Proffitt (2011, Experiment 2) argued that the bias in size estimates that they found occurred because objects appeared more graspable, and therefore smaller, when observers intended to grasp them with their right hand.

## The action-specific account: Truly a challenge to cognitive impenetrability?

As discussed by Firestone and Scholl (2016), the question of what constitutes a cognitive process is not straightforward to answer, making it difficult to determine whether action capacity should be considered truly cognitive in nature. Nevertheless, these authors suggested that action-specific effects do indeed challenge cognitive impenetrability. However, Witt (2017) argued that it is possible to accept that action-specific effects are truly perceptual without rejecting cognitive impenetrability because action-specific effects may not necessarily arise from cognitive processes. Witt (2017) also noted that Firestone and Scholl (2016) themselves rejected the strictest definition of cognitive impenetrability, which would entail that any influence on what is perceived visually by nonvisual information constitutes cognitive penetrability. For example, Firestone and Scholl (2016) suggested that multimodal effects should not be considered examples of cognitive penetrability.

Witt (2017) claimed that if top-down, cognitive influences on perception are restricted to those involving explicit knowledge affecting the visual representation of the environment, then action-specific effects should not be considered a challenge to cognitive impenetrability because these effects could be based on motor processes (Sugovic, Turk, & Witt, 2016; Witt, 2017). Similarly, Sugovic et al. (2016) suggested that “an effect based on unconscious physical abilities rather than on conscious beliefs would preserve the idea that spatial vision is cognitively impenetrable because what is known (or thought or believed) would not exert an influence on vision” (p. 1). Although it is not clear exactly what these *unconscious physical abilities* might refer to, it seems reasonable to interpret this as referring to feedback from kinaesthetic, proprioceptive, or interoceptive cues which may unconsciously specify information about the current action capabilities of the body (see Witt & Riley, 2014, for a discussion about the possible role of other sensory cues in driving action-specific effects). On this interpretation, action-specific effects may not be considered to directly challenge cognitive impenetrability.

Indeed, there is evidence for the reverse relation, namely, that motor feedback from acting, such as kinaesthetic/proprioceptive cues, can affect perceived action capacity. For example, Franchak and colleagues have shown that we update and recalibrate our perceived action capacity through acting (Franchak & Adolph, 2014; Franchak, van der Zalm & Adolph, 2010). Franchak et al. (2010) showed that participants who had prior experience of walking through apertures were subsequently more accurate at estimating whether apertures were passable. They suggested that motor feedback from performing the relevant action made participants more aware of the fit between the spatial properties of their body and that of the apertures. Extending this, it is possible that action-specific effects may result from an interaction between motor information, which may specify action capacity,^1^ and vision. This, in turn, could mean that at least some action-specific effects may be compatible with cognitive impenetrability, since they are not necessarily driven by explicit knowledge or beliefs about the action capabilities of the body, and instead may be induced by an interaction between vision and kinaesthetic/proprioceptive cues specifying action capabilities (Sugovic et al., 2016; Witt, 2017; see also Witt & Riley, 2014).

However, critically, some action-specific effects are argued to result from observers’ *beliefs* about their action capacity rather than from their actual action capacity. For example, Linkenauger, Witt, and Proffitt (2011, Experiment 2) claimed that perceived object size was scaled according to the *perceived* grasping capacity of the left and right hands. Given that they reported no *actual* difference in the grasping capacity of the hands, the only available source of the scaling effect they reported, at least according to the action-specific account, was in participant’s inaccurate *beliefs* about their grasping capacity. This claim seems to challenge cognitive impenetrability by suggesting that the critical factor in producing the reported effect was the observer’s beliefs about the action capacity of their body. The focus of this article is the reported influence of grasping capacity on perceived object size. Notwithstanding the debate as to whether action capacity itself is a cognitive factor (Firestone & Scholl, 2016), we believe that testing the claims of the action-specific account is relevant for investigating the issue of cognitive impenetrability.

## The present article: A response to Witt (2017)

Witt (2017) responded to Firestone and Scholl’s (2016) challenge by identifying four action-specific scaling effects within the existing literature and arguing that each defied all six of their pitfalls. Here, we consider in detail one of the examples that she provided, namely that one’s “ability to grasp an object affects perceived size” (p. 1011). Before discussing the reasons why we believe that grasping capacity does not, in fact, directly influence estimates of object size, we will briefly address some broader concerns with the evidence in favour of this idea.

First, we believe that the way in which grasping capacity has been manipulated in studies supporting the action-specific account is problematic. Witt (2017) claimed that it is difficult to directly manipulate grasping capacity. We disagree. From our everyday experience, it is clear that it is more difficult to grasp objects when we have cold hands, or when we wear thick gloves. Furthermore, grasping capacity can be directly manipulated in a controlled way, for example, by taping together participants’ fingers. We have employed this simple, yet effective, manipulation in our own work (Collier & Lawson, 2017a, 2017b) and, in several of our experiments, we used it to reliably reduce both perceived and actual grasping capacity, by ~2–3 cm and by ~1–2 cm, respectively.

Some of the methods used by proponents of the action-specific account to try to alter grasping capacity may be weak or unreliable. For example, Witt (2017) suggested that changing apparent hand size is a viable alternative to directly manipulating grasping capacity. However, as acknowledged by Linkenauger, Witt, and Proffitt (Linkenauger et al., 2011, b, see also Linkenauger, Leyrer, Bülthoff, & Mohler, 2013), changing visually perceived hand size risks inducing a size-contrast effect whereby objects may appear smaller when placed next to a larger hand than when placed next to a smaller hand due to visual relativity (Obonai, 1954). Any such size-contrast effect would mean that the scaling effect on perceived object size could be explained by Firestone and Scholl’s (2016) fourth pitfall, namely variation in visual features. In contrast, the visual change in hand size following taping is minimal, and so the chances of inducing a size-contrast effect is also minimised. Other studies reporting evidence in support of the action-specific account have taken advantage of the fact that right-handers both perceive their right hand as larger than their left hand, and believe that it can grasp larger objects (e.g., Linkenauger, Witt, & Proffitt, 2011). However, this effect of handedness on perceived grasping capacity is small and it does not seem to influence actual grasping capacity (Collier & Lawson, 2017a).

**Fig. 1:**
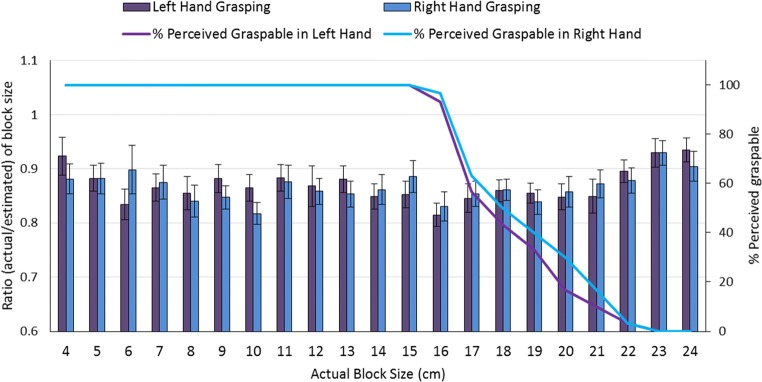
Data from Experiment 3 of Collier and Lawson (2017a), showing estimates of block size (estimated/actual, left axis and bars; note the lack of scaling effects for graspable blocks, contrary to the prediction of the action-specific account) and % of participants who thought the blocks were graspable at each block size (right axis and lines; note that these right-handed participants thought they could grasp slightly larger blocks with their right than with their left hand). Error bars show ±one standard error of the mean

Second, there are concerns with the issue of replicability and reliability within the action-specific literature. Firestone (2013) noted that many of the frequently cited findings from the action-specific account have proven difficult to replicate (e.g., De Grave, Brenner, & Smeets, 2011; Hutchison & Loomis, 2006; Woods, Philbeck, & Danoff, 2009). Our investigation into the reported effect of grasping capacity on object size began when we (Collier & Lawson, 2017a) failed to replicate Linkenauger, Witt, and Proffitt (2011, Experiment 2). In that paper, our analysis focussed on the prediction that objects within perceived maximum grasp would show effects consistent with the action-specific account. We also tested some blocks that were too big to be grasped. In Fig. 1 here, we show the size estimates for all of the blocks that we tested in Experiment 3 of Collier and Lawson (2017a), along with the percentage of our right-handed participants who thought they could grasp each block size. The action-specific account predicts that objects to be grasped by the right (rather than the left) hand should be estimated as smaller for right-handers, but only if the block is perceived as small enough to be grasped. Instead, we found no clear scaling effects regardless of block graspability. Thus, we failed to replicate Linkenauger, Witt, and Proffitt’s (2011) finding that graspable blocks are estimated as smaller for the right than the left hand, and we found no evidence that the perceived graspability of the blocks influences whether action-specific effects are found.

In the following sections, we provide evidence and arguments as to why we believe that grasping capacity does not directly influence perceived object size. We explain in detail why we disagree with the arguments given by Witt (2017) in her third case study. To do so, we provide examples from our own work to illustrate that many studies claiming to show that grasping capacity directly affects perceived object size could have an alternative explanation because they fall into at least one of three out of the six possible pitfalls outlined by Firestone and Scholl (2016). Specifically, we suggest that the results of several studies could be explained as effects arising from off-line judgement rather than online perception (Pitfall #2). We suggest that two further pitfalls, experimental demand and response bias (Pitfall #3) and low-level visual differences (Pitfall #4), could account for other effects reported in the literature. We have not directly tested the disconfirmatory claim (Pitfall #1) that objects too big to grasp do not show scaling effects. However, our failure to find that only blocks perceived as graspable show scaling effects (see Fig. 1) suggests that Witt (2017)’s argument that this effect escapes this pitfall may not be as strong as she claims. We agree with Witt (2017) that studies investigating grasping capacity and perceived object size do not generally fall into Firestone and Scholl’s (2016) Pitfall #5 (attentional effects) or Pitfall #6 (memory and recognition effects). On the basis of our findings, we conclude by arguing that perceived grasping ability does not influence perceived object size and that this third case study does not provide support for the action-specific account.

## Pitfall #2: Perception versus judgement

Firestone (2013) noted that in many experiments that have been used to support the action-specific account, it is not clear whether an effect has occurred at the level of perception (a literal change in what a person sees) or the level of judgement (an inference based on what they see). The use of indirect measures can be valuable in disentangling effects of perception from other effects. As an example of this approach, Witt (2017) cited a study where Linkenauger, Mohler, and Proffitt (2011) found an effect consistent with the action-specific account using estimated weight to indirectly assess whether differences in perceived hand size led to differences in perceived, as opposed to simply judged, object size.

However, the devil is often in the detail when distinguishing perception from judgement, and this conclusion involved a complex chain of assumptions. The predictions in Linkenauger, Mohler, and Proffitt (2011) relied on a previous finding that participants who wore magnifying goggles which globally enlarged the environment then estimated objects seen near their right hand as *smaller* (Linkenauger et al., 2010). Based on this finding and the size–weight illusion, where small objects are estimated as being heavier than larger objects of equal weight (Buckingham, 2014), Linkenauger, Mohler, and Proffitt (2011) predicted that participants wearing magnifying goggles would estimate objects seen near their right hand as *heavier*. Their participants used a pulley system to lift a reference object (a beanbag) and a test object (a basket). Right-handed participants verbally instructed the experimenter to add or remove weight from the basket to match the weight of the beanbag. Participants in the visible-hand group placed their right hand next to the beanbag, whilst participants in the hidden-hand group kept their right hand out of sight.

Linkenauger, Mohler, and Proffitt (2011) predicted that objects should be perceived as *smaller* by those in the visible-hand group than in the hidden-hand group based on the following logic. The magnifying goggles should have made everything appear larger to both groups. According to the action-specific account, in the hidden-hand group, participants should estimate beanbag size with respect to the known, remembered size of their unmagnified right hand. This would lead to larger estimates of beanbag size. In contrast, for the visible-hand group, who could see their magnified hand “the optical information specifying the size of the object is rescaled to the magnified hand” (Linkenauger, Mohler, & Proffitt, 2011, p. 1251), leading to smaller estimates of beanbag size. This, in turn, due to the size–weight illusion, should have made the beanbag feel *heavier* to the visible-hand group.

This was, indeed, what Linkenauger, Mohler, and Proffitt (2011) reported. However, we believe that there is a simpler explanation for this effect that does not involve the influence of action capacity as the action-specific account assumes. Hand size alone was not manipulated in this study. Instead, all participants wore goggles that globally enlarged the whole environment. This matters because when the environment is globally magnified, and no anchor for object size is provided in the environment, participants may estimate all objects as larger, and by extension lighter, due to the size–weight illusion. In contrast, if a salient and highly familiar object such as their own hand is visible near to the beanbag, participants may be more aware that their environment has changed, and be able to use their knowledge of the size of this object to anchor their size estimates of the beanbag. The visible-hand group could thus have used their hand as a reference to infer—in other words, judge—beanbag size, leading them to estimate the beanbags as smaller, and heavier, since they knew it had been artificially magnified and so they could discount this effect (e.g., “I know that my hand is smaller than it currently appears, so it is likely that the beanbag near it is also smaller than it currently appears”). If so, then placing a different, familiar object next to the beanbag (such as a glove) might have been just as effective as a cue to size. Linkenauger, Mohler, and Proffitt (2011) did not run this control.

Linkenauger, Mohler, and Proffitt (2011) argued that their results could not have arisen from simply using a familiar object as a size reference based on the findings of Linkenauger et al. (2010). As the results of Linkenauger et al. (2010) have rarely been discussed in detail in the literature, we think it is worth doing so here. In Experiment 1 of Linkenauger et al. (2010), participants wore magnifying goggles which globally enlarged the environment. Participants verbally estimated the size of three familiar and three unfamiliar objects, once while keeping their hand hidden out of sight and then again with their hand visible next to the objects. One group saw their right hand and the other group saw their left hand. Objects were estimated as smaller when the hand was visible, and this effect was stronger for the group who saw their right hand. Experiment 2 repeated Experiment 1 except that minification goggles were used, and only the right hand was viewed. Minified objects were estimated as larger when the right hand was visible. Linkenauger et al. (2010) argued that these effects of hand visibility could not have been driven by familiarity with the size of one’s own hand because, otherwise, reduced scaling effects should have been found for the familiar objects (because these were of known size so could, themselves, have been used as size references). However, all six of their objects were spheres, and it is not clear that their participants would have considered some to be familiar (e.g., a ping-pong ball) and others not (e.g., a styrofoam ball). Their argument that familiarity could not explain these effects would be more compelling had they measured it, for example, by having participants rate the familiarity of each object.

Familiarity effects are also relevant to the interpretation of Experiment 4 of Linkenauger et al. (2010). Here, participants estimated the size of the same six objects as in Experiment 1 except that, instead of their right hand, a pair of tongs was either present, or not, next to the objects. Before putting on the magnifying goggles, one group of participants gained experience at lifting and moving objects with the tongs (practice group), whilst a second group did not (no-practice group). The practice group subsequently estimated objects as smaller when the tongs were present than when they were not, whilst no effect was found for the no-practice group. Since tools are embodied after experience using them (e.g., Berti & Frassinetti, 2000), Linkenauger et al. (2010) interpreted this finding as showing that perceived object size was rescaled to tool size only after the tool was embodied. There is, though, once again, an alternative explanation for this finding. Before they made their size judgements, the practice group in Experiment 4 of Linkenauger et al. (2010) gained experience using the *exact pair of tongs* in the *same environment* that they were about to be tested with. Although the no-practice group had probably used kitchen tongs in their everyday lives, tongs come in different shapes and sizes and this group had not used the exact tongs that they would be tested with. Thus, in Experiment 4 only the practice group gained familiarity with the unmagnified size of the experimental tongs before they made their size judgements. The different results for the two groups could therefore, once again, have been driven by the presence of a familiar reference of known size rather than by action-specific scaling.

In Linkenauger et al. (2010) familiarity with the participant’s hand size or of tong size could explain the results of Experiments 1, 2, and 4 (and also Experiment 5, which repeated Experiment 1 but used a visual matching task instead of verbal report). In all of these experiments, participants were familiar with the size of the potential reference object *before* they wore the goggles. However, Experiment 3 of Linkenauger et al. (2010) appears to provide evidence against this account of scaling effects being modulated by using references of known size. Experiment 3 replicated Experiment 1 except that, when a hand was visible, it belonged to the experimenter rather than to the participant. Unlike Experiment 1, estimates in Experiment 3 were not influenced by hand visibility. Results of both experiments are consistent with the action-specific account because scaling effects are only predicted to occur for actions of one’s own body. However, hands vary in shape and size, and participants would probably have had little opportunity to see the unmagnified experimenter’s hand before they were tested in Experiment 3. Thus, the experimenter’s hand would not have been as effective as that of the participant’s own hand in providing a reference of known size. This could explain why size estimates in Experiment 3 were not affected by hand visibility without relying on the action-specific account. Importantly, note too that we failed to replicate this finding from Experiment 3 of Linkenauger et al. (2010) in a recent, unpublished study that we report below (see Pitfall #4: Visual Differences section) so the results of Experiment 3 may not be reliable.

Firestone (2013) suggested that the results of Linkenauger et al. (2010) could be accommodated by a modularist perspective, but he did not specifically outline how. Here, we have offered a new interpretation of these results, which suggests that the reported effects can be explained without appealing to action capacity. We suggest that neither the results of Linkenauger et al. (2010) or of Linkenauger, Mohler, and Proffitt (2011) rule out the possibility that judgements, and not perception, of object size are affected by the presence of the hand.

A similar issue concerning familiar object size may have arisen in another experiment reported in Linkenauger et al. (2013). In her third case study, Witt (2017) noted that Linkenauger et al. (2013) used virtual-reality manipulations to test whether size scaling effects predicted by the action-specific account would only occur if the apparent size of a relevant body part was altered rather than the apparent size of another familiar object. In Linkenauger et al. (2013, Experiment 3), participants saw both a familiar object (a pen) and their own hand. The pen changed size on each trial, while the participant’s hand remained constant. No scaling effects were observed. In contrast, in Linkenauger et al. (2013, Experiment 1), where only the apparent size of the participant’s hand varied, objects were estimated as smaller when placed next to a large compared to a small hand and vice versa when placed next to a large hand. However, an alternative explanation to the action-specific account of these results is that, in Experiment 3, participants may simply have preferred to use their own hand as an anchor for size estimates because it was both more stable and more familiar than the pen. A control condition where hand size and pen size are interchangeably manipulated on different trials could be valuable here. This would test whether changes in hand size, but not changes in pen size, influence object size estimations. Finding a scaling effect for both pen and hand size would suggest that a familiar object effect has occurred. Finding an effect only for hand size changes would, instead, provide evidence for the action-specific account. Without a control such as this, we cannot rule out the possibility that, in Experiment 3 of Linkenauger et al. (2013), the hand provided a convenient anchor from which the size of other objects could be inferred.

We are aware of one study that shows an indirect effect of manipulating apparent body size on perceived object size and that cannot be explained in terms of these anchoring or referencing effects. Haggard and Jundi (2009) used the rubber hand illusion to manipulate perceived hand size. In this study, the experimenter stroked both the participant’s left hand (which was hidden under a box) and a large, left glove (which was placed in view, on the box). The participant then grasped an unseen cylinder with their left hand. When the left hand and glove were stroked synchronously, the participant subsequently gave larger estimates of the cylinder weight. Haggard and Jundi (2009) suggested that the rubber hand illusion caused an increase in perceived left-hand size and this led to the unseen cylinder feeling smaller. This, in turn, meant that the cylinder felt heavier by virtue of the size–weight illusion. They concluded that people use their body representation as a reference for estimates of both positional and intrinsic properties of objects.

We agree that this finding suggests that, in this case, the left hand was used as a reference for the size of grasped cylinders. However, first, in this study, the left hand was the only size reference available. After the stroking, participants did not see the cylinder, and they did not grasp anything else. It therefore seems unsurprising that altering the perceived size of their left hand changed their perception of the cylinder. Second, if participants embodied the large glove as being their own left hand due to the synchronised stroking, and if the body is used as a reference for object size, it is not clear why this should not then have made everything visible in the environment seem smaller because it was scaled relative to the glove. Instead, for the logic of this study to work, to embody a larger left hand, the participant must have used something else as a reference to scale their left-hand representation. In other words, Haggard and Jundi (2009) argued that the left hand was used as a size reference in the weight judgement task but not during synchronised stroking where, instead, the perceived size of the left hand was assumed to be malleable. Haggard and Jundi (2009) did not discuss in detail the mechanism by which participants were assumed to embody a larger left hand. It could be argued that, for their right-handed participants, their right hand, which they used to respond with, acted as a visual size reference relative to the left glove because right-handers prefer to use the right rather than the left hand as a reference. This possibility could be tested, for example, by having only the experimenter’s right hand visible rather than that of the participant. However, no such control condition was run by Haggard and Jundi (2009). Third, although the results of this study suggest that body size can be used as a reference for estimating object size, this is not the same as the claim of the action-specific account that it is a person’s action capabilities that matter for size scaling. Body size and action capacity are often tightly related, but they should not be conflated. For instance, two people with the same hand size may differ in their grasping capacity due to differences in flexibility or grip strength.

In summary, unlike Witt (2017), we do not believe that the use of estimated weight as an indirect measure for perceived size (Linkenauger, Mohler, & Proffitt, 2011) has provided convincing evidence in favour of the argument that grasping capacity influences perceived object size. This is because the scaling effects found in Linkenauger, Mohler, and Proffitt (2011; see also Linkenauger et al., 2010) could, instead, arise from providing a salient and familiar cue of known size (such as the participant’s hand), which participants then use to anchor their object size estimates. We have further suggested that a similar issue with familiar size could have occurred in Linkenauger et al. (2013), since the results of their control experiment using a pen that changed in size could have been confounded by the presence of the participant’s own hand, which did not change in size.

## Pitfall #3: Experimental demand and response bias

The action-specific account has recently been under pressure to demonstrate that effects consistent with it are not the result of experimental demand characteristics (e.g., Collier & Lawson, 2017a, 2017b; Durgin et al., 2009; Firestone, 2013; Firestone & Scholl, 2014, 2016). In our own work, we have shown that at least some studies claiming to show an influence of grasping capacity on perceived object size can be explained by experimental demand and response bias. After we (Collier & Lawson, 2017a) failed to replicate Linkenauger, Witt, and Proffitt (2011, Experiment 2), we sought to understand why. A critical difference between Collier and Lawson (2017a) and Linkenauger, Witt, and Proffitt (2011, Experiment 2) is that, in the latter study, participants were asked on each trial whether they thought the blocks were graspable. This was done to ensure that participants intended to act on the blocks, which is regarded as critical to obtaining action-specific scaling effects (Linkenauger, Witt, & Proffitt, 2011; Witt et al., 2005). In Collier and Lawson (2017a), we did not do this because we were concerned that explicitly asking participants about the graspability of the blocks on each trial could unintentionally introduce task demands or response biases. We reasoned that the dimensions of graspable-to-ungraspable and small-to-large could be conceptually linked or conflated by participants (e.g., Walker, 2012). We therefore hypothesised that judging graspability immediately before estimating size on every trial could have led to size estimates being biased by the immediately preceding graspability judgements in Linkenauger, Witt, and Proffitt (2011, Experiment 2). This conflation could have led to graspable blocks being estimated as smaller. In Experiment 3 of Collier and Lawson (2017b), we tested whether this methodological difference could explain the discrepancy in results between Linkenauger, Witt, and Proffitt (2011, Experiment 2) and Collier and Lawson (2017a) by asking participants to rate the difficulty of grasping a block before estimating its size. In contrast to the null results that we reported in Collier and Lawson (2017a), where such context or conflation effects were controlled for, now, in Collier and Lawson (2017b), participants estimated objects they grasped in their taped hand as larger than objects they grasped in their untaped hand. This suggests that the scaling effect reported by Linkenauger, Witt, and Proffitt (2011, Experiment 2) likely also reflected a response bias arising from asking participants about two conceptually linked dimensions in quick succession. If so, then the scaling effect was not a true perceptual change.

In a different experiment, we used a cover story to try to reduce the experimental demand involved with grasping in this size-estimation task (Collier & Lawson, 2017a, Experiment 5). The inspiration for this manipulation came from previous studies showing that action-specific effects are often not found when experimental demand is minimised by using a cover story (e.g., Durgin et al., 2009; Firestone & Scholl, 2014). Here, we asked participants to visually match the size of blocks they had just grasped with their left hand, their right hand, and after the fingers of one of their hands were taped together. To ensure that participants intended to act, and knew that we were interested in their grasping behaviour, we told them repeatedly and explicitly that we were recording whether they could grasp the blocks. In addition, on each trial, they had to actually grasp the block both before and after estimating its size. Critically, though, to control for demand characteristics associated with doing these two tasks on the same trial, we also told them a cover story that the grasping task and the size estimation task were providing data for separate studies, and that they were only doing both tasks together because of time constraints. Perceived action capacity changed as expected: Our right-handed participants believed that they could grasp bigger objects in their right hand than their left hand, and bigger objects in their untaped hand than their taped hand. However, crucially, we found no differences in their estimates of object size depending on which hand they intended to grasp the block with, and whether that hand was taped. If the influence of grasping capacity was truly perceptual, our cover story manipulation should not have worked and, according to the action-specific account, objects should have been estimated as larger for the left hand and larger still for the taped hand. These results demonstrate that scaling effects in object grasping studies can be eliminated when experimental task demands are minimised.

## Pitfall #4: Visual differences

Many studies investigating the influence of grasping capacity on estimates of object size manipulate apparent grasping capacity by changing the visually perceived size of the hand (e.g., Linkenauger et al., 2013; Linkenauger, Witt, & Proffitt, 2011, Experiment 3). In her third case study, Witt (2017) acknowledges the possibility that the effects obtained in such studies could be caused by visual differences between conditions because “visual differences in hand size are key to obtaining these effects” (p. 1013). Manipulations such as taping, which alter participants’ grasping capacity with little effect on hand size, should help to rule out explanations in terms of visual differences because they largely avoid this fourth pitfall. However, when we have used a taping manipulation, we have found no influence of grasping capacity on estimated object size when we have controlled for conflation (Collier & Lawson, 2017a, 2017b). This means that it is possible that visual differences could account for at least some of the scaling effects reported in other studies which manipulated hand size using magnification and minification in order to vary grasping capacity (e.g., Linkenauger et al., 2013; Linkenauger, Witt, & Proffitt, 2011).

For example, in Linkenauger, Witt, and Proffitt (2011, Experiment 3), participants estimated the grasping capacity of their dominant hand while it was, and was not, magnified and they also estimated the size of objects placed near their hand. Objects were estimated as smaller when placed near to the magnified hand. Linkenauger, Witt, and Proffitt (2011, Experiment 3) claimed that this demonstrated a scaling of object size by perceived grasping capacity. They discussed, but ultimately rejected, the explanation that visual differences, in the form of size-contrast effects, could explain their result because they found that only objects that were perceived as graspable showed the predicted scaling effect. They claimed that a size-contrast explanation would produce contrast effects across all stimulus sizes, regardless of perceived graspability.

Linkenauger et al. (2013) also tested whether size-contrast effects could explain their finding, in their first experiment, that objects were estimated as smaller when participants’ hands were enlarged using virtual reality. In their second experiment they manipulated the size of the hands of a virtual avatar and found no effect on estimated object size. Thus, the combined results of Linkenauger, Witt, and Proffitt (2011, Experiment 3) and Linkenauger et al. (2013, Experiments 1 and 2) seem to suggest that size-contrast effects cannot explain action-specific scaling effects.

**Fig. 2:**
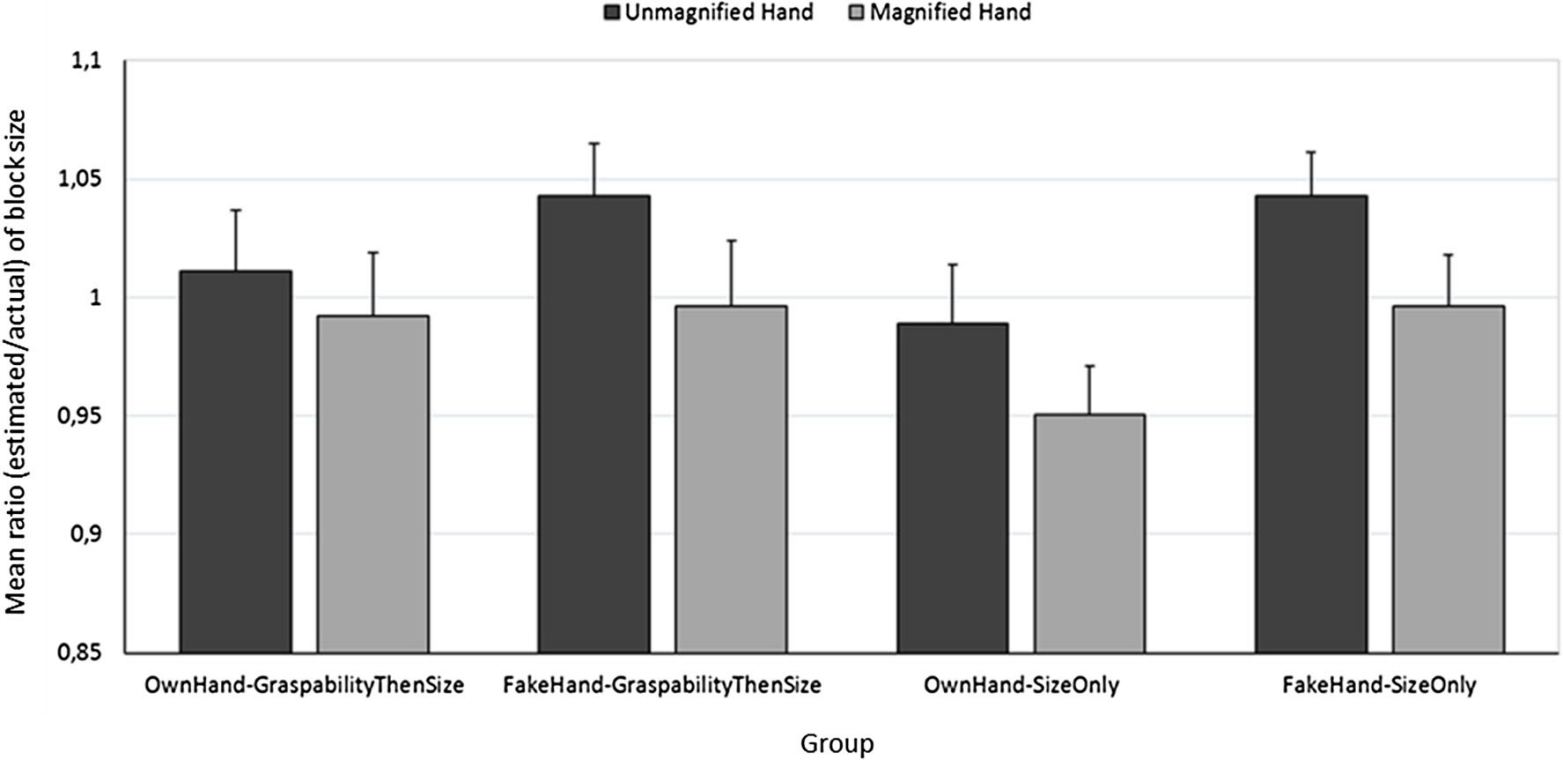
Mean ratio (estimated/actual) of block size for the unmagnified and magnified hands in each of the four groups. A ratio of 1 represents perfect accuracy. Error bars show one standard error of the mean

However, this may not be the case. For example, in a recent study, we used the same magnification manipulation as Linkenauger, Witt, and Proffitt (2011, Experiment 3). We tested four groups of 16 participants. The first group decided whether their right hand could grasp a block that was placed next to it and then they visually matched the size of the block. A second group did the same two tasks but a fake, plastic right hand replaced their own right hand and they were asked if it could grasp the block if it could move. A third and fourth group were matched to these two groups, but they only did the size matching task. All groups made estimates while the visible hand, whether their own or fake, was both magnified and unmagnified (in separate subblocks with a counterbalanced order). We tested whether, as predicted by the action-specific account (Linkenauger et al., 2013; Linkenauger, Witt, & Proffitt, 2011), scaling effects would occur only for the first, OwnHand-GraspabilityThenSize group since only here did participants both make estimates when their own hand was visible (rather than a fake hand) and intend to act on the block (as they were asked about grasping it). Contrary to these predictions, we instead found that blocks were estimated to be smaller when they were seen next to a magnified (compared to an unmagnified) hand in all four groups (see Fig. 2). Details of the method and results of this previously unpublished experiment are given in the supplementary material.

## Summary and conclusion

Firestone and Scholl (2016) outlined six pitfalls which, they claimed, can explain nearly all effects which claim to demonstrate a direct, top-down influence of cognition on perception. They argued that if an effect falls into just one of these pitfalls, it should not be considered a true demonstration of cognitive penetrability. An important test of this approach are the results used to support the action-specific account, which claims that what we perceive is scaled according to our action capacity (Proffitt & Linkenauger, 2013; Witt, 2011, 2017). Witt (2017) responded to Firestone and Scholl (2016) by claiming that at least four action-specific effects withstand all six of their pitfalls.

The third case that Witt (2017) discussed was the influence of apparent grasping capacity on perceived object size. In this article, we have challenged Witt’s claim with respect to this effect. We argued that grasping capacity (whether actual or perceived) does not directly influence perceived object size. To this end, we critically examined the claims Witt (2017) made in her third case study, and we provided new interpretations of previous results claiming to support the action-specific account. We noted that many of the studies cited by Witt (2017) in her third case study did not directly manipulate grasping capacity but, instead, altered perceived hand size using, for example, handedness effects, magnification, or virtual reality, and that this may have introduced confounds. We also noted that many action-specific effects, including the purported effect of grasping capacity on estimates of object size, have been difficult to replicate. Then we provided empirical evidence from our own work which suggests that studies claiming to demonstrate this effect in fact appear to fall prey to at least one, and possibly several, of the pitfalls outlined by Firestone and Scholl (2016). It is important to emphasise that we are not arguing that the entire action-specific account is necessarily incorrect. Rather, we are arguing that this specific case that Witt (2017) claimed withstands all six pitfalls does not provide strong evidence in favour of the account.

To summarise our findings across several experiments:

1. We failed to replicate the finding that the greater perceived grasping capacity of the right compared to the left hand for right-handers increases estimates of the size of objects that are intended to be grasped by the right hand (Collier & Lawson, 2017a, Experiments 2 & 3).
2. We found no effect of directly manipulating perceived grasping capacity, by taping together the fingers of a hand, on estimates of the size of objects to be grasped by the taped hand (Collier & Lawson, 2017a, Experiments 4 & 5; 2017b).
3. We only found scaling effects consistent with the action-specific account under restricted, nonecological circumstances, namely, when estimates of perceived object size could be conflated with perceived action capacity because participants were asked to estimate their ability to grasp an object immediately before being asked to estimate its size (Collier & Lawson, 2017b).
4. We found that using a convincing cover story to minimise experimental demand characteristics and conflation effects eliminated the effect of grasping capacity on estimates of object size (Collier & Lawson, 2017a, Experiment 5).
5. We found that visual differences resulting from, for example, magnification, could explain the apparent influence of grasping capacity on estimates of object size (new data reported in this paper; see also Supplementary Materials).

In conclusion, many studies claiming to show an effect of grasping capacity on perceived object size fall into at least one of the pitfalls outlined by Firestone and Scholl (2016). Furthermore, the results from our own studies do not suggest that grasping capacity influences perceived object size. We argue that the scaling effects that have been reported in this case need not be interpreted as revealing a true perceptual change caused by altering action capacity, and therefore that these effects do not challenge cognitive impenetrability.

## Electronic supplementary material

ESM 1 (DOCX 163 kb)

## Footnotes

1 However, there is some evidence against this alternative account. For example, in our studies (Collier & Lawson, 2017a, 2017b; see also Collier & Lawson, 2018) we taped together the fingers of one hand, restricting both actual and perceived grasping capacity. Since participants actually acted in our experiments, they received motor feedback about the restricted grasping capacity of their taped hand relative to their untaped hand. If action-specific effects were driven by such motor feedback, we should have found effects consistent with the action-specific account in our experiments. However, we found no indication that changes in either actual or perceived grasping capacity influenced estimates of object size. Thus, for this action-specific effect, we found no evidence that it was driven by motor processes.
