# Supplementary material for: Getting a grasp on action-specific scaling: A response to Witt (2017)
Source: Psychon Bull Rev. 2018 Sep 20;26(1):374–84. doi: 10.3758/s13423-018-1511-0 (PMC6425068; doi:10.3758/s13423-018-1511-0)
Supplement: Supplementary file 1 — (DOCX 163 kb) [file 13423_2018_1511_MOESM1_ESM.docx]

### SUPPLEMENTARY MATERIAL

### Method and results of a study investigating whether estimates of object size scale according to the predictions of the action-specific account of perception.

### We tested four groups of 16 participants. The first group decided whether their right hand could grasp a block that was placed next to it and then they visually matched the size of the block. A second group did the same two tasks but a fake, plastic right hand replaced their own right hand and they were asked if this hand could grasp the block if it could move. A third and fourth group were matched to these two groups but they only did the size matching task. All groups made estimates while the visible hand (whether their own or fake) was both magnified and unmagnified. We tested whether effects consistent with the action-specific account would occur. The action-specific account predicts that blocks should be estimated as smaller when placed next to the magnified hand, but only by the first group. Only here was both the participant's own hand size manipulated and the participant had to consider if the blocks were graspable. Thus participants in this first group both made estimates when their own hand was visible (rather than a fake hand) and intended to act on the block (as they were asked whether they could grasp tit). Similar predictions were tested by Linkenauger et al. (2013, Experiment 2) and by Linkenauger, Witt and Proffitt (2011, Experiment 3) respectively. Contrary to these predictions, we found an effect of magnification in all four groups, see Figure 2.

### Method

### Participants

Sixty-four participants^[[1]](#footnote-1)^ (mean age = 24.6 years, 18 males) were recruited for this experiment. Participants either volunteered or were given course credit for their time. All but one self-reported as right handed. Ethical approval for this experiment was granted by the relevant local ethics committee at the University of Liverpool.

### Stimuli, design and procedure

The stimuli were five foamboard square blocks (0.5 cm thick) with sides of 5, 10, 20, 30 and 40 cm. Two blocks (5 cm, 10 cm) were always graspable, two blocks (30 cm, 40 cm) were always too large to be grasped, and one block (20 cm) was considered to be near the threshold of perceived maximum grasping capacity for the right hand (this was 18cm for a similar population of participants in Experiment 3 of Collier and Lawson, 2017a).

A magnification box and a control box were constructed with sides of 28 cm × 21 cm and a depth of 9.8 cm. The top of the magnification box was made from a transparent sheet which made a hand placed inside the box appear around 50% larger than the same hand outside the box. The top of the control box was made from clear plastic so a hand seen inside this box was not magnified.

One of the two boxes was placed on a table in front of the participant, on their right side. Participants sat at the table and either their own right hand or a fake hand was placed inside the box, see Figure 1. On each trial, they closed their eyes while the experimenter placed one of the blocks flat on the table beside the box. Participants then opened their eyes to look at the block.

Sixteen participants were assigned to each of four groups: OwnHand-GraspabilityThenSize, FakeHand-GraspabilityThenSize, OwnHand-SizeOnly, or FakeHand-SizeOnly. Those in the OwnHand groups viewed their right hand, while those in the FakeHand groups kept their right hand out of sight and viewed a fake, plastic right hand (lifelike colour, width at widest point = 8.7 cm, length at longest point = 14.2 cm). The participant's left hand was hidden from view throughout testing. On each trial all participants first did a relative length task, then, for the GraspabilityThenSize groups only, they did a graspability task. Finally all participants ended the trial with a size estimation task. These three tasks are now described in turn.

All participants were first asked how similar in length the block appeared relative to the visible hand as it appeared inside the box. Responses were made on a scale of 1 (very different) to 10 (the same length). This *relative length task* ensured that all participants looked at both the visible hand and the block, so they could have used the hand as an anchor for object size. We also reasoned that this relative length task could reduce demand characteristics by providing a reason why a hand was prominently visible throughout the experiment.

All participants also estimated the width of the block in the *size estimation task*. Here, the experimenter slowly pulled out a tape measure, with the numbers facing away from the participant. The participant verbally guided them by saying “bigger” or “smaller” until they thought that the visible length of tape measure matched the width of the block. The tape measure was pulled out vertically to minimise the use of landmark matching strategies. The experimenter looked away from the tape measure while pulling it so that they could not see participants’ estimates until the participant said “stop”. The experimenter encouraged the participant to make minor adjustments to their response before they said stop. The participant then closed their eyes and the block was exchanged for the next block. The visible hand was not removed from the box between trials.

The size estimation task immediately followed the relative length task for the SizeOnly groups. The GraspabilityThenSize groups did an additional *graspability task* on each trial, after the relative length task and before they did the size estimation task*.* In the graspability task they made a forced (yes/no) choice verbal decision as to whether they thought the block could be grasped by the visible hand. They were told to make their graspability judgements based on how the hand appeared in the box and by imagining placing the thumb of the hand on one side of the block and any other finger on the opposite side. The FakeHand groups were also asked to imagine that the hand could move.

Participants did two sub-blocks each comprising five trials: one with the visible hand inside the magnifying box and the other with the visible hand inside the non-magnifying, control box. Sub-block order was counterbalanced across participants within each group. Within each sub-block each size of block was presented once and block order was randomised for each participant. For the OwnHand groups, the experimenter measured the width and length of participant's right hands after they had completed the relative length and size estimation tasks. Participants laid their hand flat on the table with the fingers together, and the experimenter used the measuring tape to measure the width across the widest part of their hand, and the length from their wrist to the end of their middle finger.


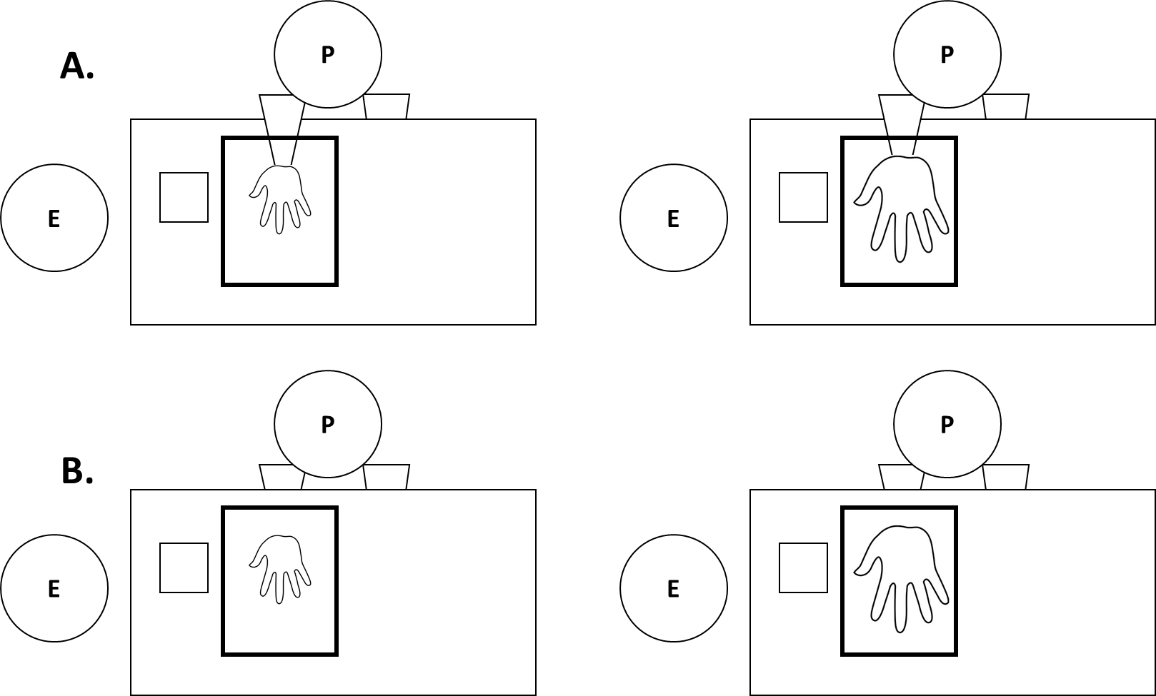


*Figure 1:* The experimental set-up for the OwnHand (A, top) and FakeHand (B, bottom) groups for a non-magnifying, control box trial (left) and a magnifying box trial (right). The experimenter (E) and participant (P) are represented by circles next to a rectangle representing the table with a square block stimulus on it and the control or magnifying box. Participants in the OwnHand conditions kept their left hand only out of sight, while participants in the FakeHand conditions kept both of their hands out of sight.

### Results and Discussion

The mean unmagnified size of the visible hand in the OwnHand groups was similar to that of the fake hand (width 8.7cm, length 14.2cm), being 9.6cm and 17.1cm respectively for the OwnHand-SizeOnly and 9.7cm and 17.2cm respectively for the OwnHand-GraspabilityThenSize group.

For each block for each participant, perceived block size was calculated as a ratio of actual block size by dividing estimated by actual block size. These ratios were used as the dependent variable in a repeated measures ANOVA with magnification (Magnified/Unmagnified) as a within-participants factor, and visible hand (Own/Fake) and task (SizeOnly/GraspabilityThenSize) as between-participants factors. This revealed a main effect of magnification such that blocks were estimated as smaller when placed next to the magnified hand (m = 0.98, se = 0.01) than the control, unmagnified hand (m = 1.01, se = 0.01), *F*(1, 60) = 16.882, *p* < .001, η_p_^2^ = .22. There were no other significant effects. For the main effects of hand, *F*(1, 60) = 0.347, *p* = .6, η_p_^2^ = .006; and of task, *F*(1, 60) = 0.048, *p* = .8, η_p_^2^ = .001. For the interactions: magnification × hand, *F*(1, 60) = 0.261, *p* = .6, η_p_^2^ = .004; magnification × task, *F*(1, 60) = 1.576, *p* = .2, η_p_^2^ = .003; hand × task, *F*(1, 60) = 2.367, *p* = .2, η_p_^2^ = .04; magnification × hand × task, *F*(1, 60) < 0.001, *p* = .9, η_p_^2^ < .001, see Figure 2.


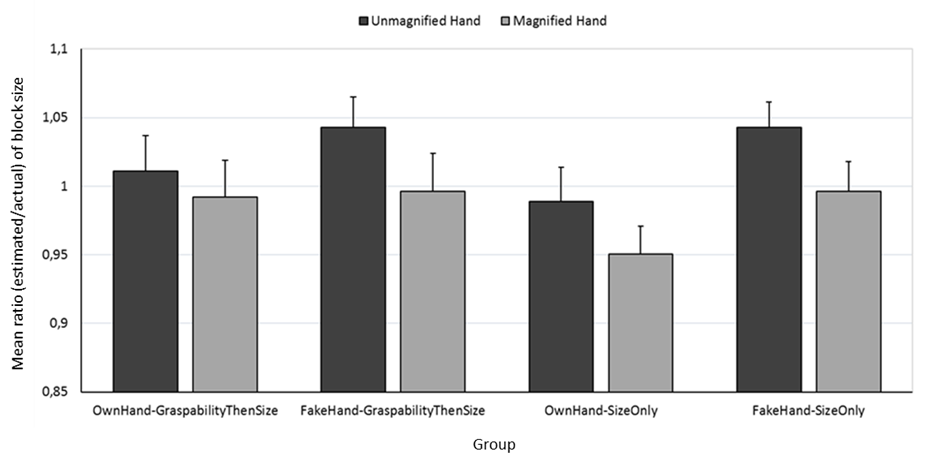


*Figure 2:*  Mean ratio (estimated/actual) of block size for the magnified and the control, unmagnified hands in each of the four groups. The action-specific account predicts that only the first group should estimate the blocks as smaller when placed next to the magnified compared to the unmagnified hand. Error bars show one standard error of the mean.

The analysis we used here differs from that used by Linkenauger, Witt and Proffitt (2011, Experiment 3), where the data were split by whether participants judged the blocks to be graspable or not. Since only two of the four groups in this experiment rated graspability we chose to include all the data in a single analysis. Table 1 shows that the effect of magnification was in the same direction for most block sizes across all four groups.

**Table 1**
Mean difference between size estimates with magnified and unmagnified hands for each block size for each group. Positive numbers indicate that blocks were estimated as smaller when placed next to the magnified hand than the unmagnified hand, and vice versa for negative numbers.

| Block Size | OwnHand  Graspability  ThenSize | FakeHand  Graspability  ThenSize | OwnHand  SizeOnly | FakeHand  SizeOnly |
| --- | --- | --- | --- | --- |
| 5 | 0.059 | 0.065 | 0.069 | 0.094 |
| 10 | 0.053 | 0.034 | 0.024 | 0.081 |
| 20 | 0.003 | 0.045 | 0.042 | 0.006 |
| 30 | 0.031 | -0.012 | 0.031 | 0.031 |
| 40 | -0.052 | 0.003 | 0.027 | 0.022 |

In summary, in the present study, there was no indication that the reduced size estimates for blocks placed next to a magnified hand were confined to the OwnHand-GraspabilityThenSize group, contrary to the prediction based on the action-specific account. Instead, this group had a smaller absolute difference between the magnified and unmagnified conditions than the other three groups tested (see Figure 2).

1. Sixty-seven participants were tested, but the data from three participants was not analysed (two from the OwnHand-SizeOnly group and one from the OwnHand-GraspabilityThenSize group). Two of these participants correctly guessed the purpose of the study during debrief, and one was replaced because of experimenter error. [↑](#footnote-ref-1)
